# Supplementary material for: A wearable electrostimulation-augmented ionic-gel photothermal patch doped with MXene for skin tumor treatment
Source: Nat Commun. 2024 Jan 26;15:762. doi: 10.1038/s41467-024-45070-z (PMC10817919; doi:10.1038/s41467-024-45070-z)
Supplement: Supplementary file 1 — Supplementary Information [file 41467_2024_45070_MOESM1_ESM.pdf]

Supplementary Information

A wearable electrostimulation-augmented ionic-gel photothermal patch doped with MXene for skin tumor treatment

Xingkai Ju, [1, 2]Jiao Kong, [1, 2]Guohua Qi*,[1,] Shuping Hou, [1, 2]Xingkang Diao[1,2] Shaojun Dong[1,2,] Yongdong Jin*[1,2,3]

[1]State Key Laboratory of Electroanalytical Chemistry, Changchun Institute of Applied Chemistry, Chinese Academy of Sciences, Changchun 130022, China.

[2]School of Applied Chemistry and Engineering, University of Science and Technology of China, Hefei 230026, China.

[3]Guangdong Key Laboratory of Biomedical Measurements and Ultrasound Imaging, School of Biomedical Engineering, Shenzhen University Medical School, Shenzhen University, Shenzhen 518060, China.

Email: ghqi@ciac.ac.cn; ydjin@ciac.ac.cn;

Experimental section

Materials

Ti_3AlC_2 (200 mesh, 98%) and Lithium fluoride were brought from Macklin. Acrylic acid (AA), N, N'-Methylenebis(acrylamide) (MBAA), and Acrylamide (AAM) were brought from Aladdin. 1-Ethyl-3-methylimidazolium (EMIES) was brought from Shanghai Yuanye. 2-Hydroxy-4'-(2-hydroxyethoxy)-2-methyproplophenone (I_{2959}) was brought from TCI. 2′, 7′-dichlorofluorescein diacetate (DCFH-DA), and Calcein-AM/PI were brought from Sigma-Aldrich. The 3-(4, 5-dimethylthiazol-2-yl)-2,5-diphenyltetrazolium bromide (MTT), JC-1 assay kit, and dimethyl sulfoxide (DMSO) were bought from Key-Gen BioTech. The antibiotic solution was purchased from VivaCell, Shanghai, China. The fetal bovine serum (FBS) was purchased from Inner Mongolia Opcel Biotechnology Co., Ltd. Anti-γ H2A.X (phospho S139) antibody

(ab81299) was purchased from Abcam. The Cy3 labeled goat antirabbit IgG (H + L) antibody (K1209) was bought from APE×BIO. ATP content detection kit was purchased from Solarbio. Haematite, eosin and TUNEL cell apoptosis detection assay kits were obtained from Thermo Fisher. All the solutions were prepared through the ultrapure water which was obtained using a Millipore Milli-Qwater purification system (Billerica, MA), with an electric resistance >18.25 MΩ.

Instruments

JEM-2100F transmission electron microscope (TEM) and an XL30 ESEM scanning electron microscope (SEM) were used to characterize the morphology of 2D material nanoflakes. SEM samples were prepared on the substrate of the AAO membrane to identify their structures. XRD analysis was performed using a Bruker D8 Advanced diffractometer with filtered Cu Kα radiation (λ = 0.154 nm). AFM imaging was performed using a Dimension Icon (Bruker). X-ray photoelectron spectroscopy (XPS) measurements were carried out on Escalab 250Xi, Thermo Fisher Scientific. The UV-Vis absorption spectra were taken using a Lambda 750 spectrophotometer (Perkin-Elmer). The Fluke infrared thermometer (TiS40) was performed for infrared imaging. The microplate reader was purchased from Tecan, which was used to detect the absorbance of formazan for assessing cell cytotoxicity. The electrochemical measurements were performed on the CHI660C electrochemical workstation. Viscoelastic measurements were conducted on a strain-controlled rheometer (ARES G2, TA, USA) using 8mm parallel plates. Stabilized voltage supply (HY3005B) which purchased from HuaYi Instrument Co., Ltd was used for electrostimulation. The tensile test was performed on a universal testing machine (AGS-X 100N, SHIMADZU, Japan) with a stretched deformation rate of 100 mm min^{-1}. An inverted microscope (Leica DMI6000B, Germany) with an external double channel optical system was used for imaging.

Preparation of MXene nanosheets

Typically, the MXene nanosheets were prepared based on the work reported, previously[1]. 1.0 g of LiF was dissolved in 10 mL HCl solution (9 M) completely by stirring in the Teflon reactor, and then the Ti_3AlC_2 (0.5g) was slowly added into the

solution. Afterward, the Teflon reactor was heated in a water bath at 35 °C with 400 rpm stirring for 24 h. To achieve the neutral solution, the product was collected and washed with ultrapure water by many times. Finally, the neutral solution was centrifuged for at 1369 g for 0.5 h to obtain a dark green MXene solution, MXene nanosheets of which were obtained by freeze-drying for further use.

SEM characterization

To characterize the pore structure inside the ionic gels, the ionic gels were immersed in deionized water for five minutes, then embrittled in liquid nitrogen and then sputtered with platinum using an XL30 ESEM scanning electron microscope for imaging.

Calculation of the photothermal transduction efficiency

Following the report[2], the total energy input and dissipation from the system can be written as:

$$\sum_i m_i C_{p,i} \frac{dT}{dt} = Q_{in,Mxene} + Q_{in,sys} - Q_{out} \quad (1)$$

where the i terms $m_iC_{p,i}$ are products of mass and heat capacity of system components, including MXene solution and cuvette. T is the solution temperature, $Q_{in,\ MXene}$ is the energy absorbed by MXene nanosheets, $Q_{in,\ sys}$ is the energy inputted to the sample cell in the absence of MXene nanosheets, and Q_{out} is heat dissipation of the system.

The heat input term $Q_{in,sys}$ can be expressed as:

$$Q_{in,Mxene} = I(1 - 10^{-A_{808}})\eta \quad (2)$$

where I is incident laser power in W, η is the photothermal transduction efficiency, and A_{808} is the absorbance of the MXene at specific wavelength 808 nm.

The system heat dissipation term Q_{out} is linear with system temperature:

$$Q_{out} = hS(T - T_{surr}) \quad (3)$$

where h is heating transfer coefficient, S is the exposed surface area of the cuvette, and T_{surr} is the ambient temperature of the surroundings.

At system equilibrium, solution temperature reaches T_{max}, and the heat input and output are balanced:

$$Q_{in,Mxene} + Q_{in,sys} = Q_{out} = hS(T - T_{surr}) \quad (4)$$

When light is turned off, the heat input terms become zero, Eq. 1 becomes

$$\sum_i m_i C_{p,i} \frac{dT}{dt} = -Q_{out} = -hS(T - T_{surr}) \tag{5}$$

rearranging Eq. 5 would give

$$dt = -\frac{\sum_i m_i C_{p,i}}{hS} \frac{dT}{(T - T_{surr})} \tag{6}$$

and by integration, Eq. 6 becomes

$$t = -\frac{\sum_i m_i C_{p,i}}{hS} ln \frac{T - T_{surr}}{T_{max} - T_{surr}} \tag{7}$$

A system time constant τ_s is defined as:

$$\tau_s = \frac{\sum_i m_i C_{p,i}}{hS} \tag{8}$$

and a dimensionless term θ is introduced:

$$\theta = \frac{T - T_{surr}}{T_{max} - T_{surr}} \tag{9}$$

Substituting Eq. 8 and 9 into Eq. 7 gives:

$$t = -\tau_s ln\theta \tag{10}$$

Thus, the time constant for system heat transfer τ_s can be determined by linear regression of the time data vs. negative natural logarithm of θ.

$Q_{in,sys}$ can be measured directly as:

$$Q_{in,sys} = hS(T_{max,H_2O} - T_{surr}) \tag{11}$$

and Eq. 4 can be rewritten as:

$$Q_{in,Mxene} = I(1 - 10^{-A_{808}})\eta = hS(T_{max,H_2O} - T_{surr}) \tag{12}$$

With τ_s = 305.92 s obtained by fitting (Figure S), m is 0.8 g and the C is 4.2 J/g, hS is calculated to be 10.98 mW/°C. Substituting I = 0.75 W, A_{808} = 2.673, T_{max}-$T_{max, H2O}$ =19.74 °C into Eq. 12, the photothermal transduction efficiency is calculated to be about 30 %.

Detection of intracellular ROS

To detect the ROS variation within cells after different treatments, the 2’,7’-dichlorofluorescin (DCFH-DA) solution (10 μM) was used, which is the specific dye for ROS detection[3]. Typically, the cells were washed using the PBS three times before treatments and stained using the DCFH-DA in an incubator at 37 °C for 30 min. Subsequently, the cells were cleaned and treated with different methods. The ROS levels within cells were observed using the fluorescence microscope after each

treatment for 30 min.

Real-time Polymerase Chain Reaction (PCR)

To further examine the gene expression after the PES treatment, real-time PCR was used to detect six genes including cellular pyroptosis markers (Caspase-3, GSDME, IL-1β) and apoptotic marker (Bax, c-Jun, Cyt-c).[4] First, the cells were stimulated with PES conditions as the experimental group, while untreated cells were used as the control group. After that, total mRNA was obtained from commercial assay kits and reverse transcribed into an equal volume of cDNA according to a reverse transcription kit. Amplification was performed using real-time fluorescence quantitative PCR. Three parallel samples were applied for each experimental group. The primer sequences of each gene used are listed in Supplementary Table 1.

Histological Analysis

After fifteen days of observation, all mice were executed and dissected, and the tumor was weighed and photographed. The tissues (heart, liver, lung, kidney, and spleen) and tumors of the mice were fixed using 4% paraformaldehyde solution, embedded in paraffin, cut to 5 μm thickness, and subjected to histological analysis using hematoxylin and eosin (H&E) staining. Immunohistochemical staining was performed using the KI-67 antibody as a marker of cell proliferation.

Blood chemistry analysis

First, whole blood was drawn from the eye socket of tumor-bearing mice from different groups. Healthy mice served as the control group. Blood samples were analyzed for routine blood analysis by the Automatic Blood Analyzer using a protocol approved by Jilin ADICON Clinical Laboratory. Inc.

Supplementary Figures

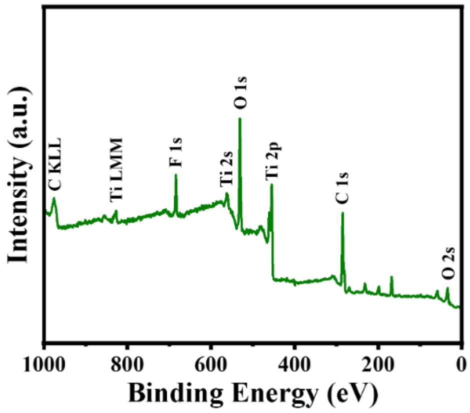

Supplementary Figure 1 │ The XPS spectrum of the $Ti_3C_2T_x$.

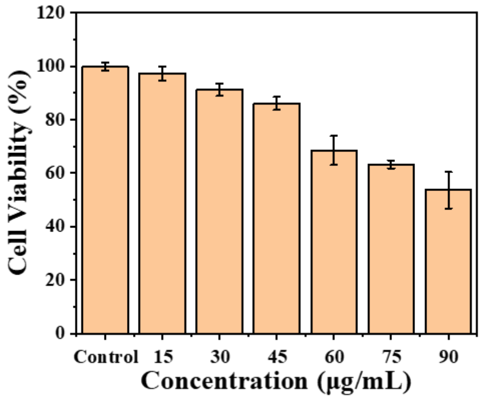

Supplementary Figure 2 │ Cell viability of B16F10 cells incubated with different concentration of MXene after irradiation by 808 nm laser for 10 min.

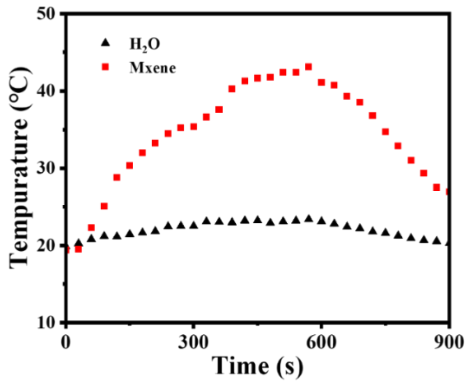

Supplementary Figure 3 │ Temperature variation curves of MXene solution and water irradiated by laser for 600 s, followed by natural cooling with laser light turned off.

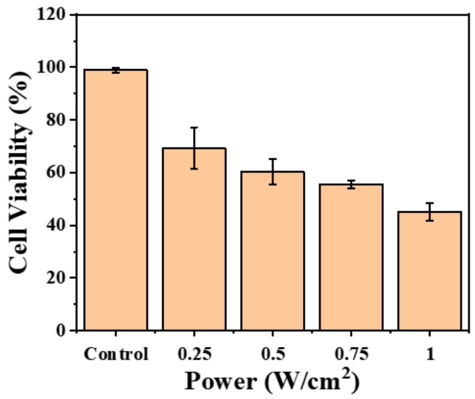

Supplementary Figure 4 | The cell viability of cells after incubation with MXene medium (45 μg/mL) irradiated by 808 nm laser at different power levels for 10 min.

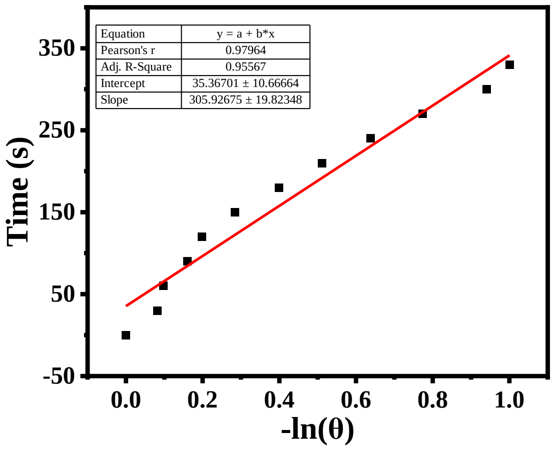

Supplementary Figure 5 | The time constant of heat transfer measured by cooling linear regression.

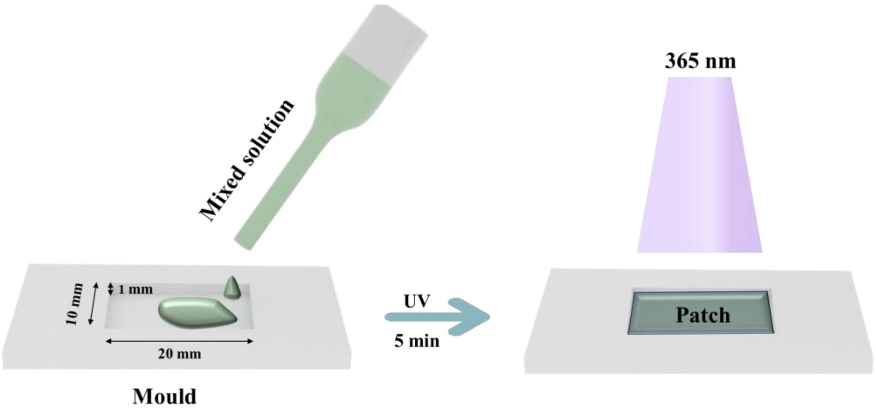

Supplementary Figure 6 | The schematic preparation process of the eT-patch.

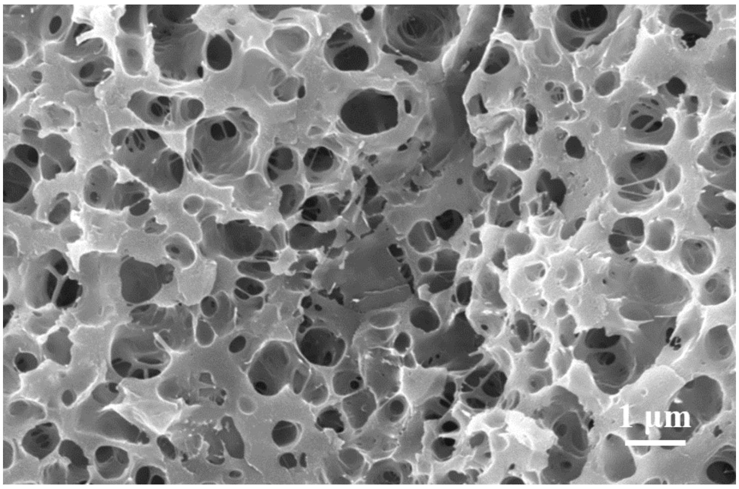

Supplementary Figure 7 | Typical SEM image of the internal structure of the eT-patch.

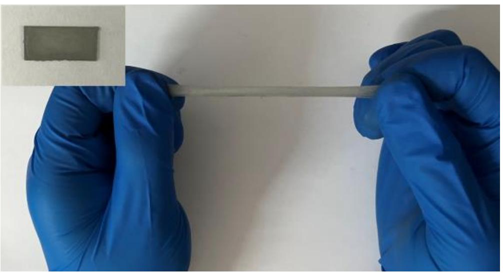

Supplementary Figure 8 | Optical photo of the stretched eT-patch (top left inset is the unstretched patch).

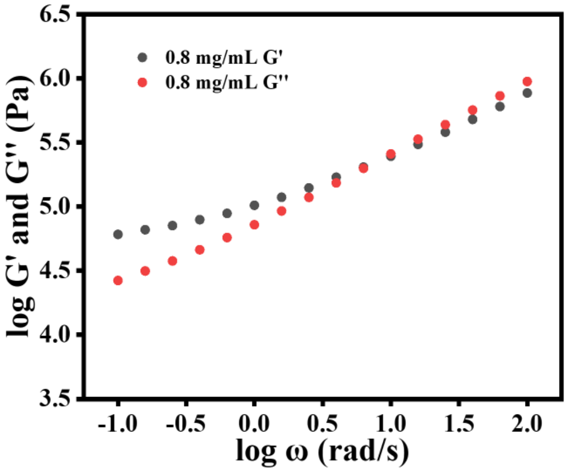

Supplementary Figure 9 | Rheological frequency sweeps of the eT-patch. (Temperature: 37 ℃)

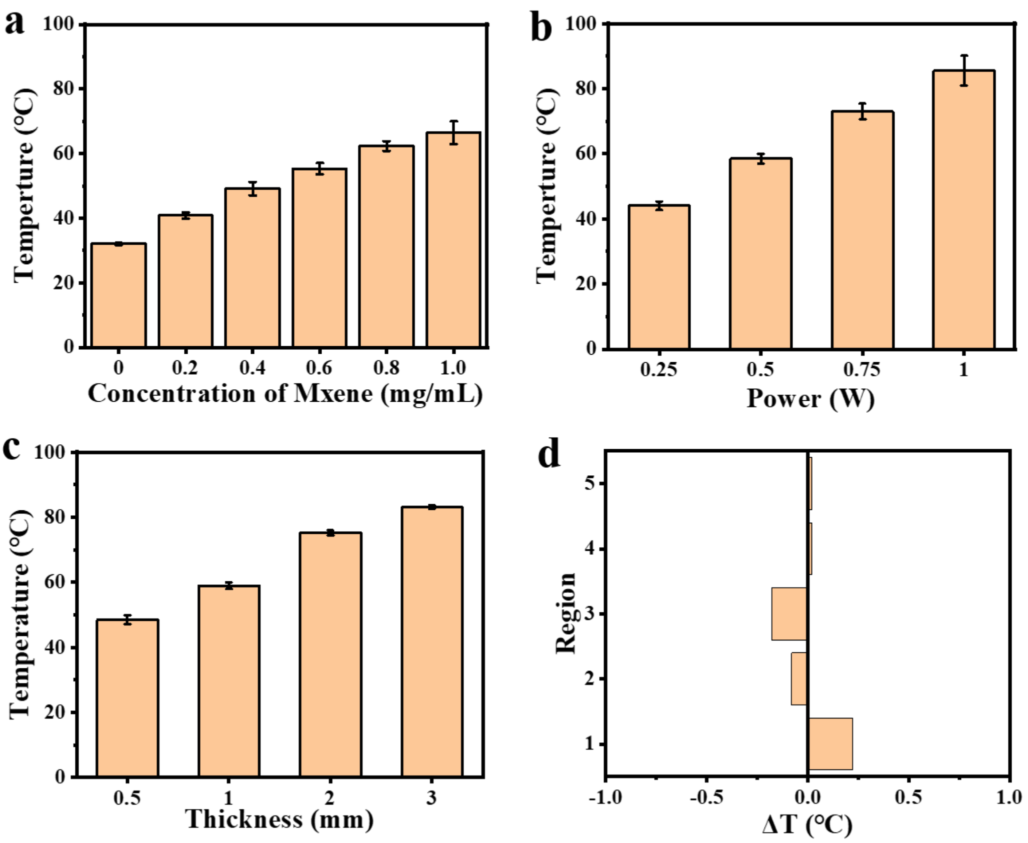

Supplementary Figure 10 │ (**a**) Temperature variation of ionic gels patches doped with MXene under different concentrations irradiated with 808 nm laser at 0.5 W/cm^2 power for 10 min. (**b**) The temperature variation of eT-patches contained with MXene concentration of 0.8 mg/mL irradiated at different powers for 10 min. (**c**) The temperature variation of eT-patches doped with MXene concentration of 0.8 mg/mL under different thicknesses, irradiated at 0.5 W/cm^2 for 10 min. (**d**) The temperature variation of a eT-patch measured at different regions under 0.5 W/cm^2 laser irradiation for 10 min.

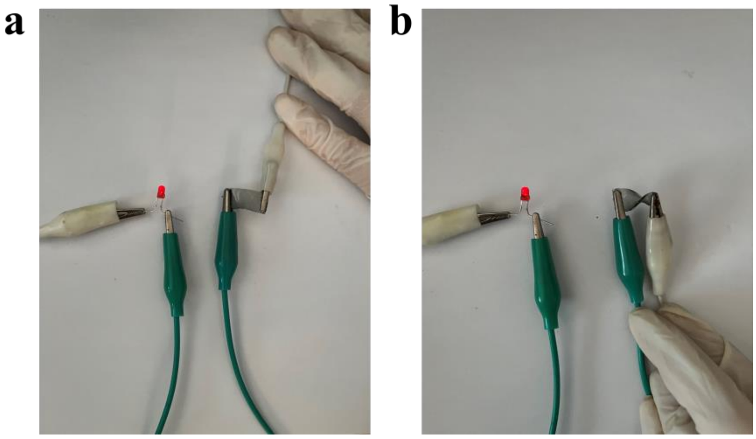

Supplementary Figure 11 │ (**a**) The current passing through the patch lights up a small LED bulb. (**b**) The current passes through the twisted patch to light up the small bulb.

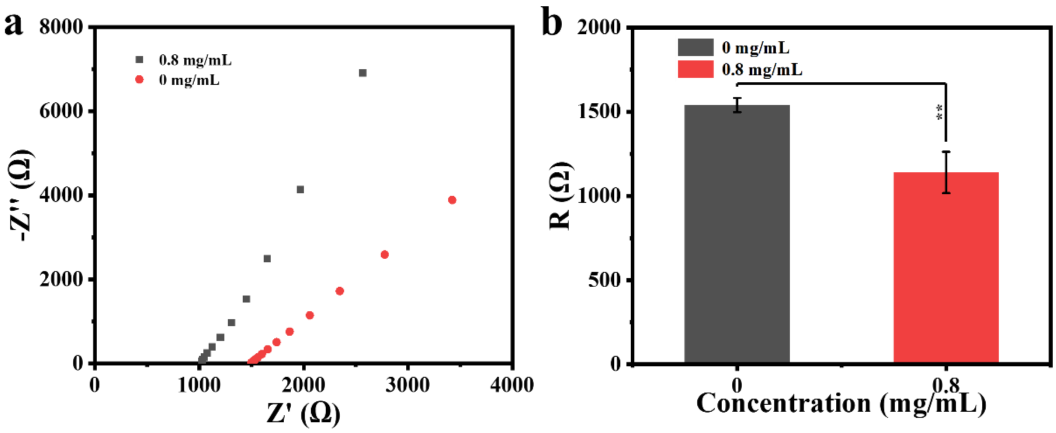

Supplementary Figure 12 │ (a) Zoomed image of Nyquist plots of the patch without or with MXene doping (0.8 mg/ mL) at the high frequency region. (b) The impedance values of the patches without or with MXene doping (0.8 mg/ mL). P values calculated by two-tailed t-tests. (**$p < 0.01$)

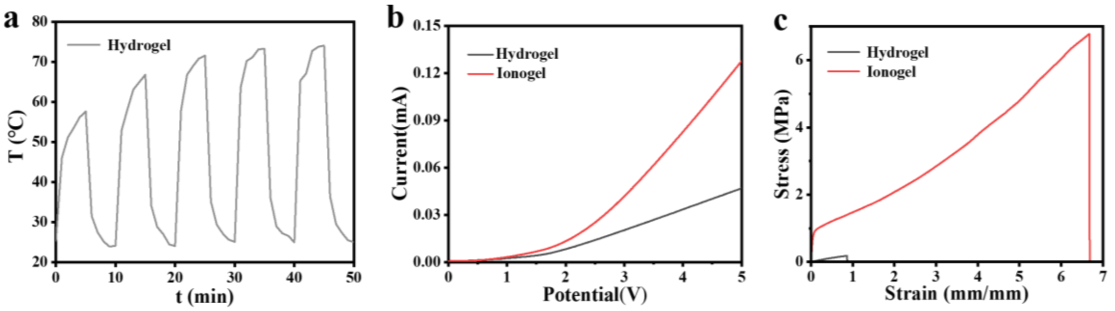

Supplementary Figure 13│ (a) Cycling temperature profile for the poly (acrylamide-co-acrylic acid) hydrogel doped with 0.8 mg/mL MXene under 808 nm laser irradiation at 0.5 W/cm^2. (b) Current-voltage curves at 5 V of the ionic gel and hydrogel patches, respectively, doped with MXene. (c) Typical stress-strain curves of the ionic gel and hydrogel patches, respectively, doped with MXene.

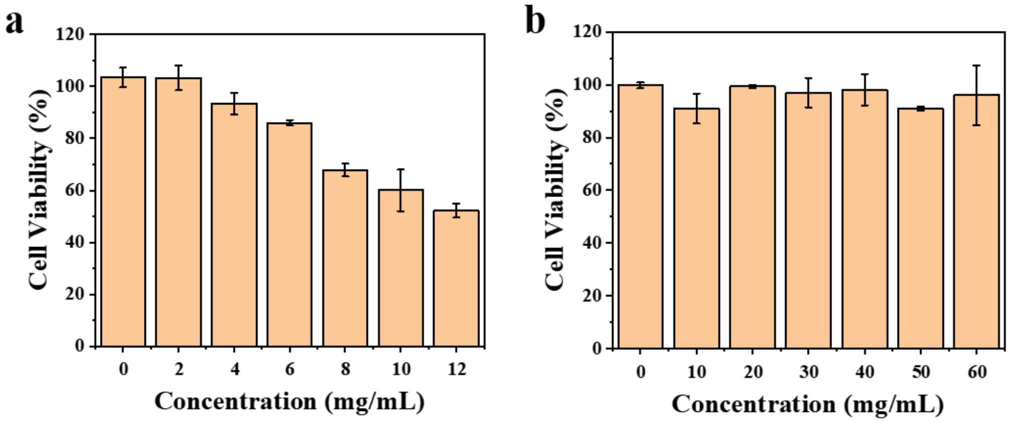

Supplementary Figure 14 │ Cell viability of B16F10 cells incubated in different concentrations of gel extracts (**a**) for 24 h and (**b**) for 10 min detected using the MTT assay.

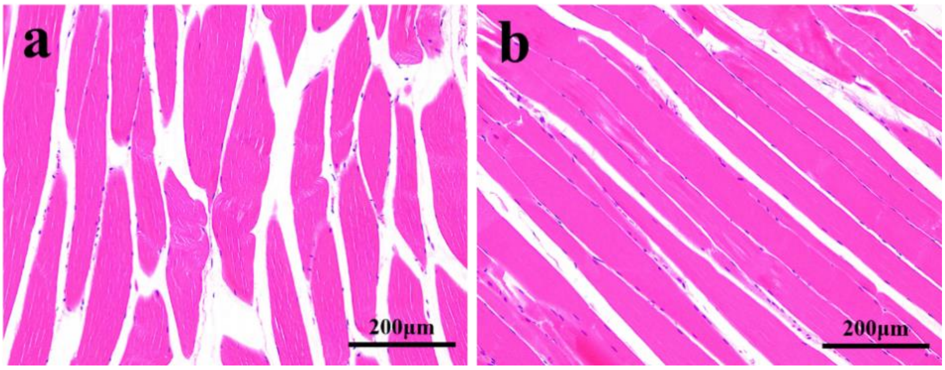

Supplementary Figure 15 │ The H&E staining of pork tissue before (**a**) and after (**b**) the eT-patch pasting.

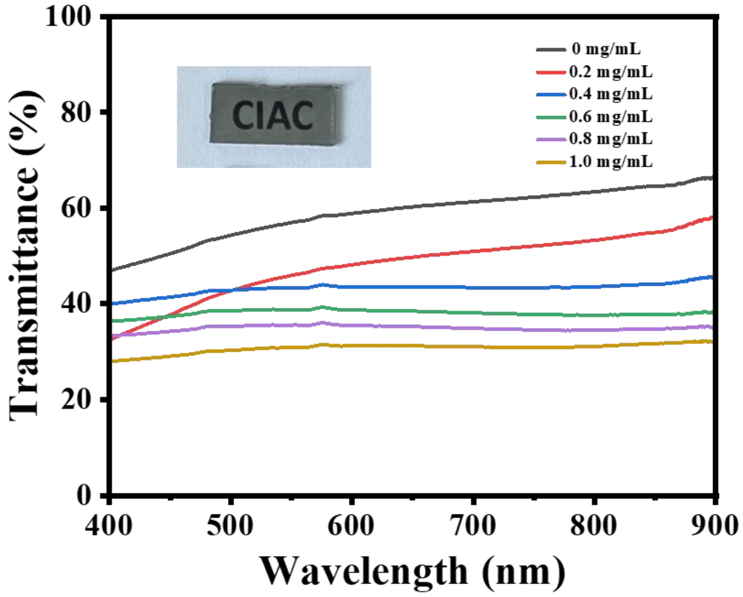

Supplementary Figure 16 │ The transmittance of ionic gels patch doped with different concentrations of MXene (The inset on the top left is an optical photograph of the gel at a MXene concentration of 0.8 mg/mL).

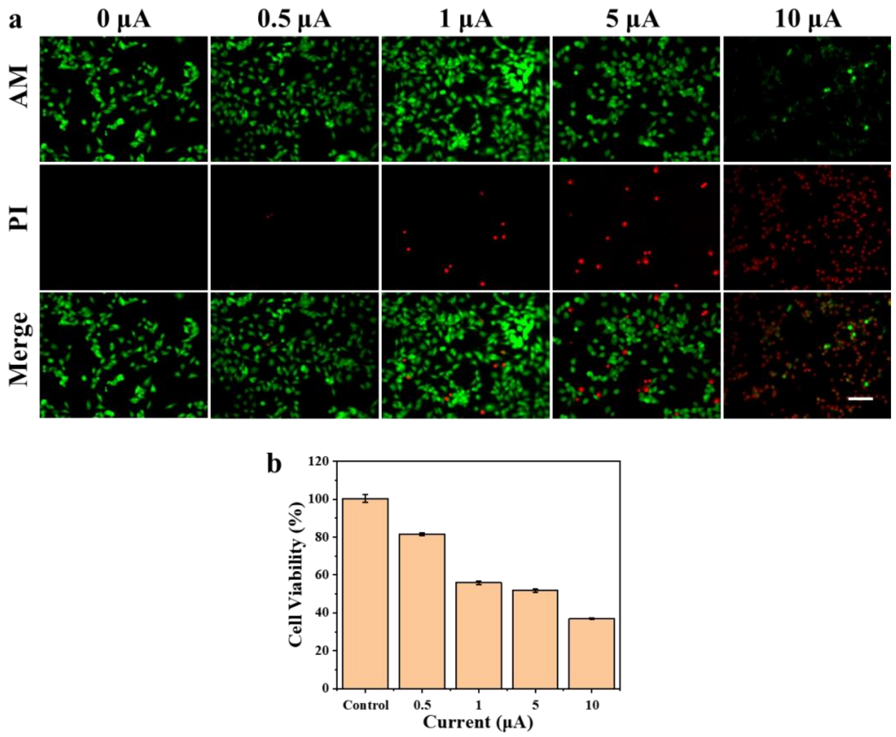

Supplementary Figure 17 │ (**a**) Fluorescence imaging of B16F10 cells after stimulation with different currents for 10 min. The scale bar is 100 μm. (**b**) The cell viability of B16F10 cells after the treatments under different currents detected using MTT assay.

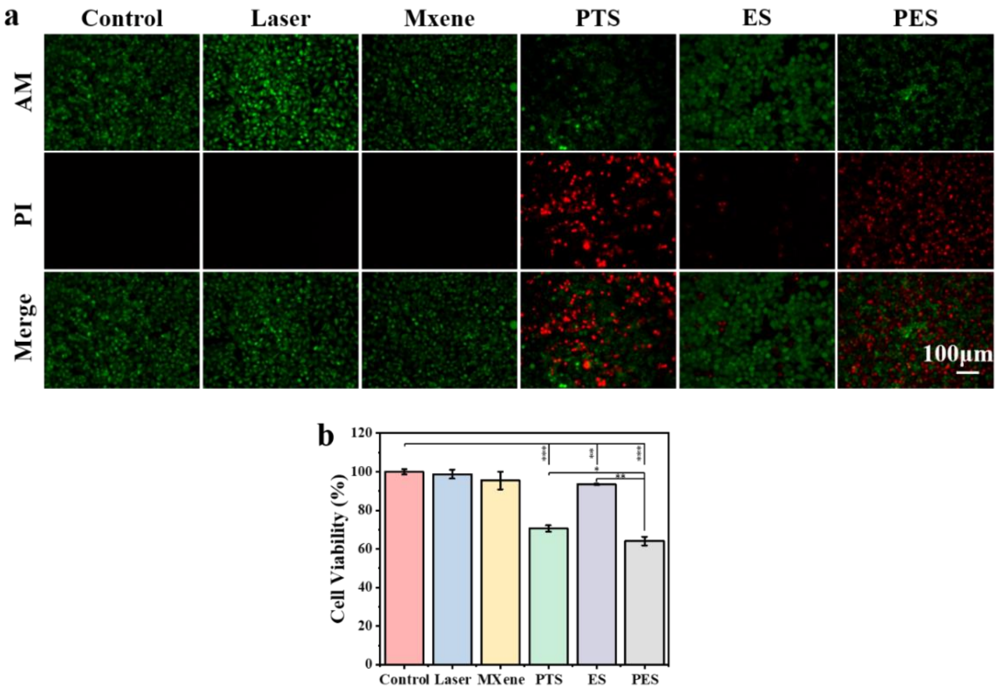

Supplementary Figure 18 │ (**a**) Live/dead staining images of normal L929 cells after the different treatments. The scale bar is 100 μm. (**b**) Cell viability of L929 cells after the different treatments. P values were calculated by two-tailed t-tests. (*p < 0.05, **p < 0.005, ***p < 0.0001).

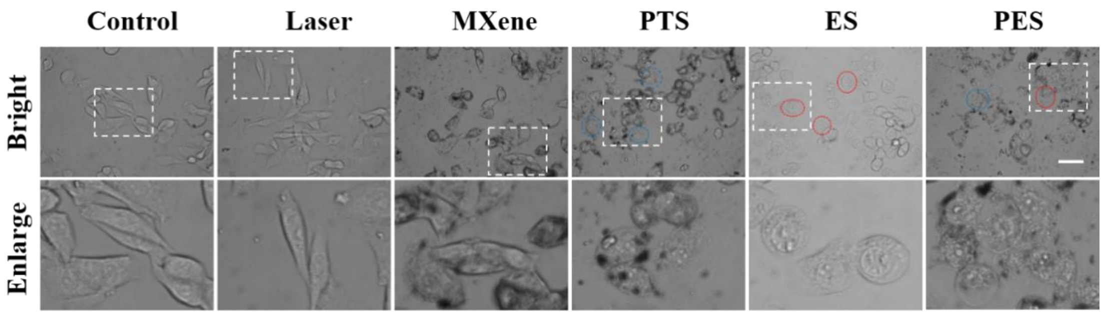

Supplementary Figure 19 │ Bright-field images of B16F10 cells after the treatment under different conditions. The scale bar is 50 μm. Red circle representing pyroptosis, the bule circle representing apoptosis, the white aquare representing the enlarged area.

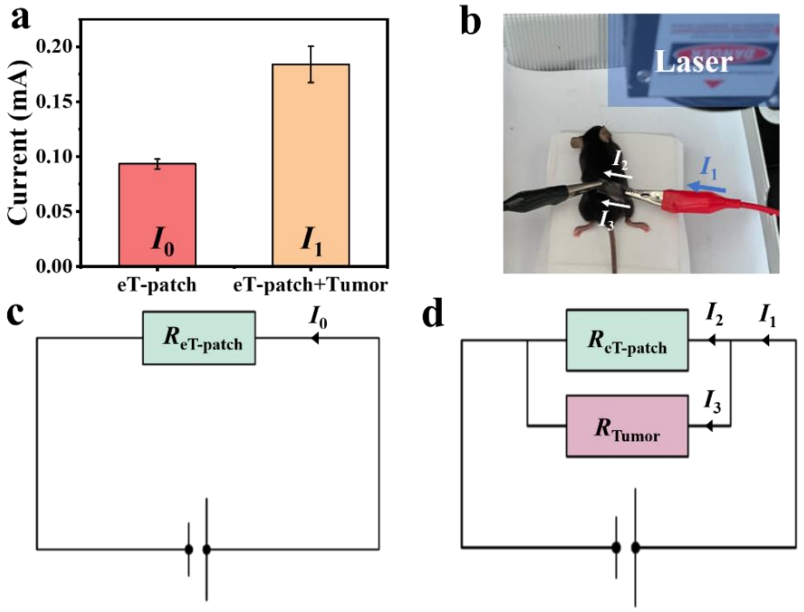

Supplementary Figure 20 │ (**a**) The current flowing through the free-standing eT-patch and the one covering on the tumor, measured at 5 V. (**b**) The photo of experimental setup showing direction of current flow during the treatment. (**c**) Circuit diagram with eT-patch only. (**d**) Circuit diagram with eT-patch covered on the tumor.

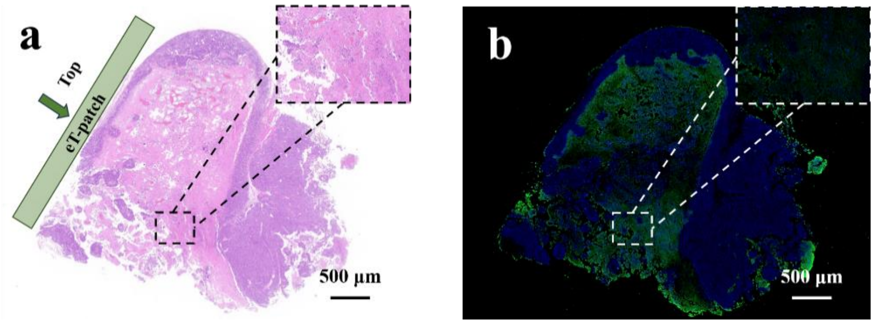

Supplementary Figure 21 │ H&E staining (**a**) and TUNEL staining (**b**) images of tumors after the PES treatment under 0.5 W/cm^2 irradiation and 100 μA stimulation for 10 min.

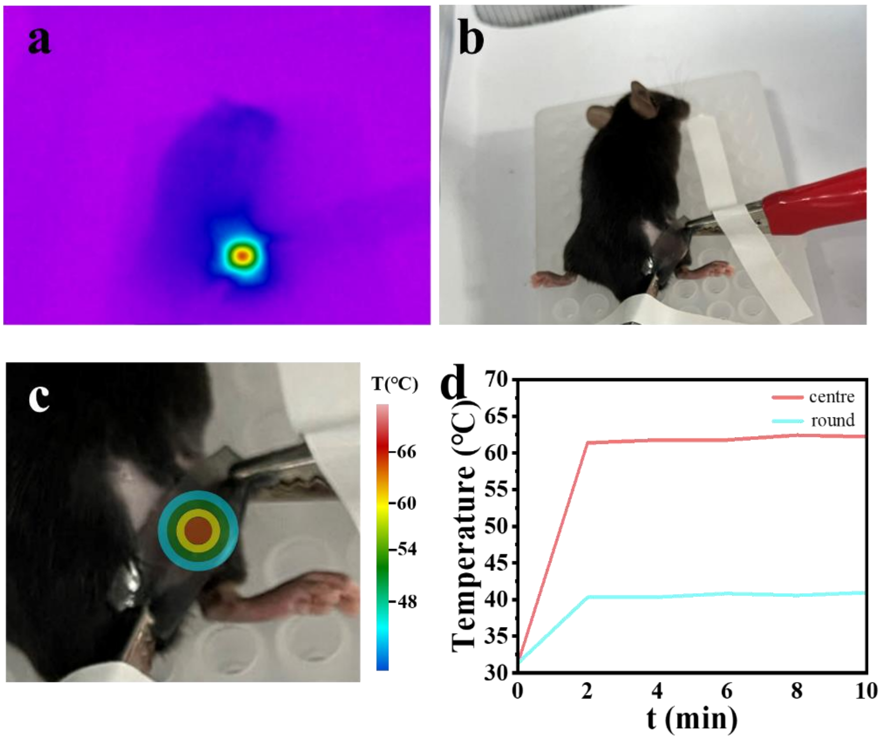

Supplementary Figure 22 │ (**a**) Thermal images of tumor-bearing mice under PES treatment. (**b**) The optical photograph of tumor-bearing mice under PES treatment. (**c**-**d**) Temperature distribution diagram and corresponding temperature variation curves of tumor-bearing mice under PES treatment.

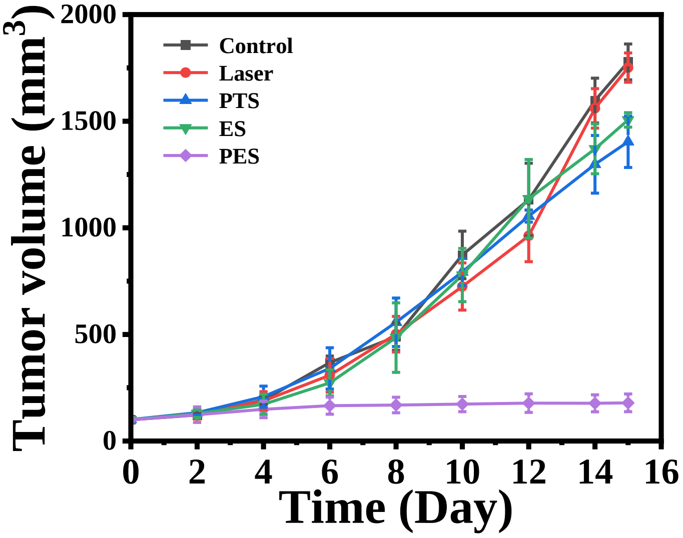

Supplementary Figure 23 │ **Changes of tumor volume in different groups of mice.**

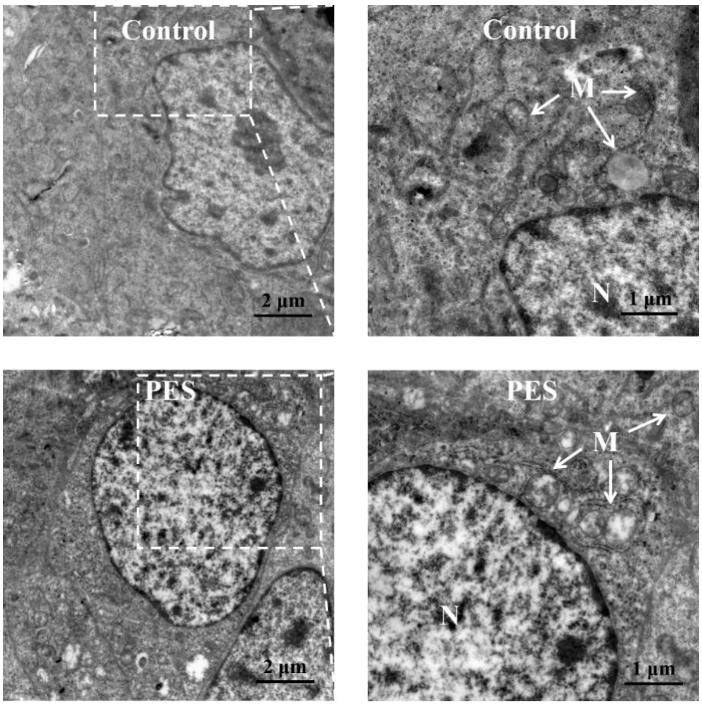

Supplementary Figure 24 | TEM images of cells in control and PES groups after treatment. “M” represents mitochondria, “N” represents cell nucleus.

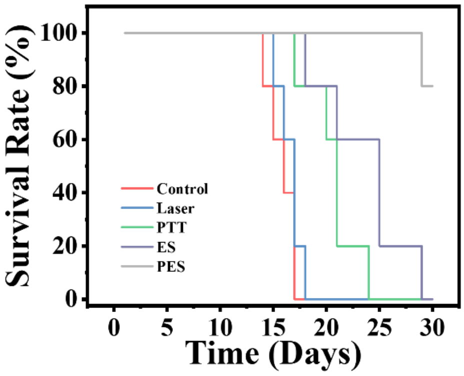

Supplementary Figure 25 | Long-term survival rates of tumor bearing mice subjected to different treatments.

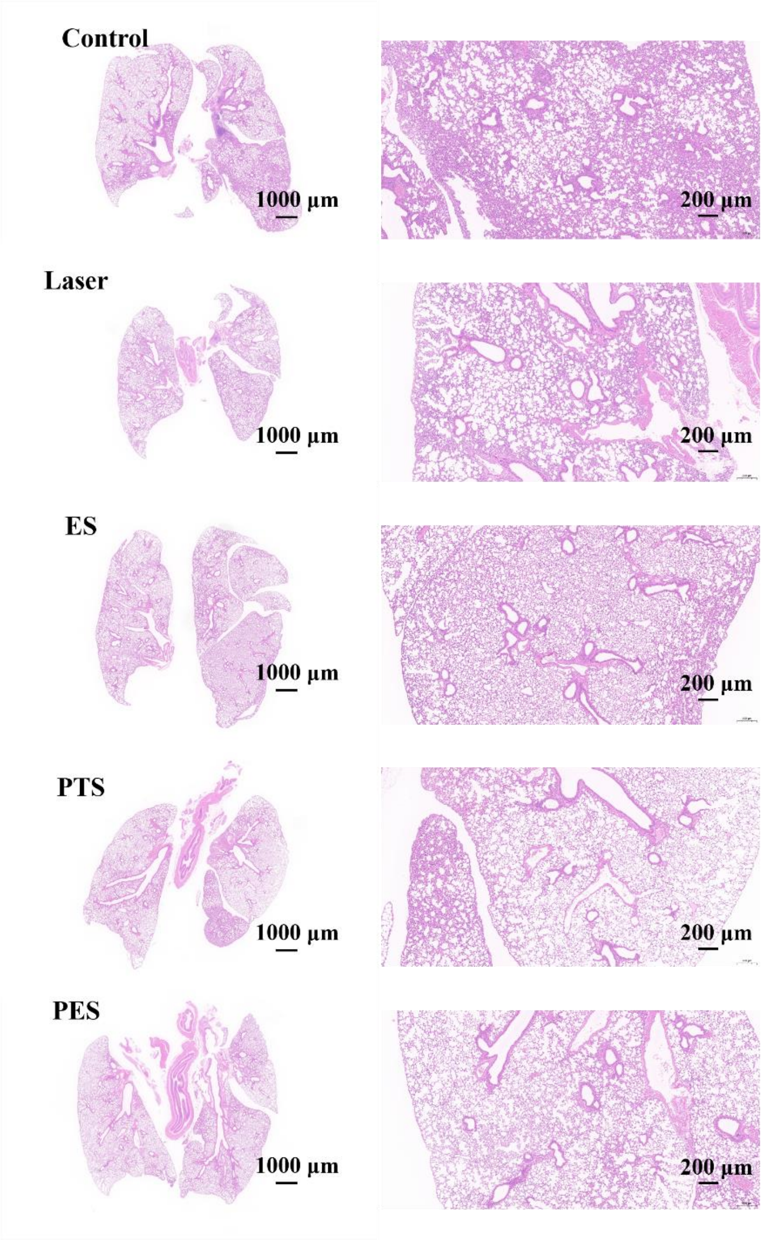

Supplementary Figure 26 │ H&E staining images of lungs from tumor-bearing 20-day mice with different treatments.

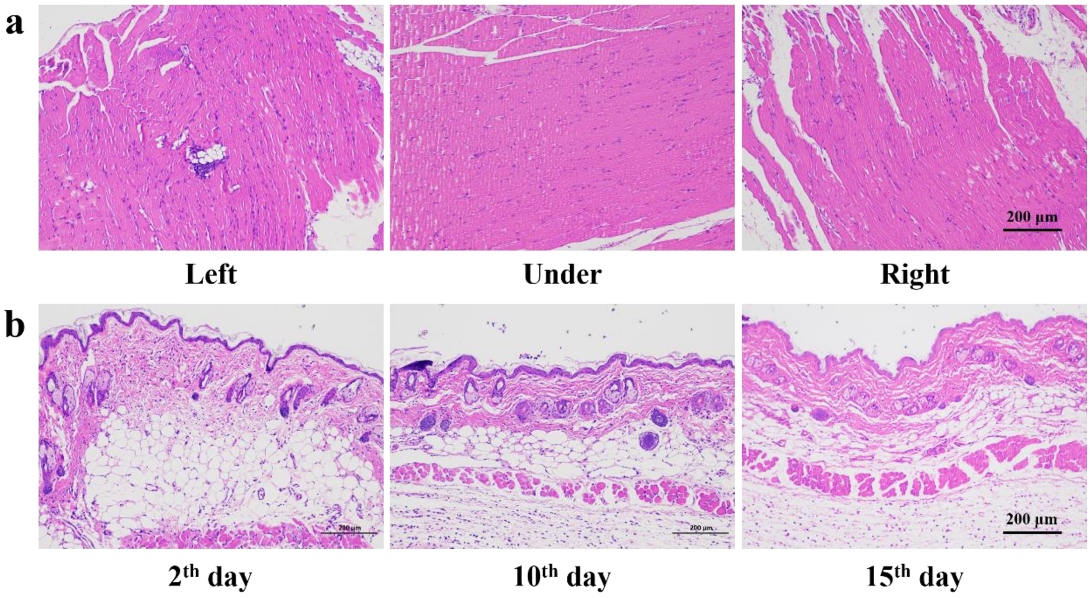

Supplementary Figure 27 │ (**a**) H&E staining images of muscle around tumor tissue after the PES treatment. (**b**) H&E staining images of skin at different days of treatment site.

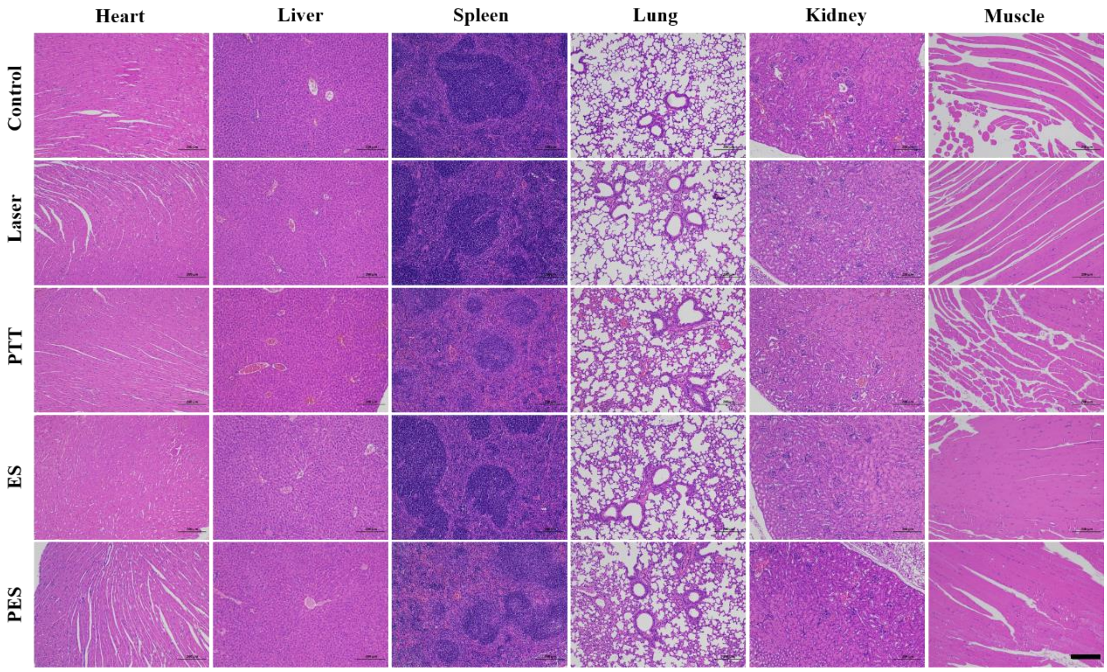

Supplementary Figure 28 │ H&E staining of major organs of mice after the various treatments. The scale bar is 200 μm.

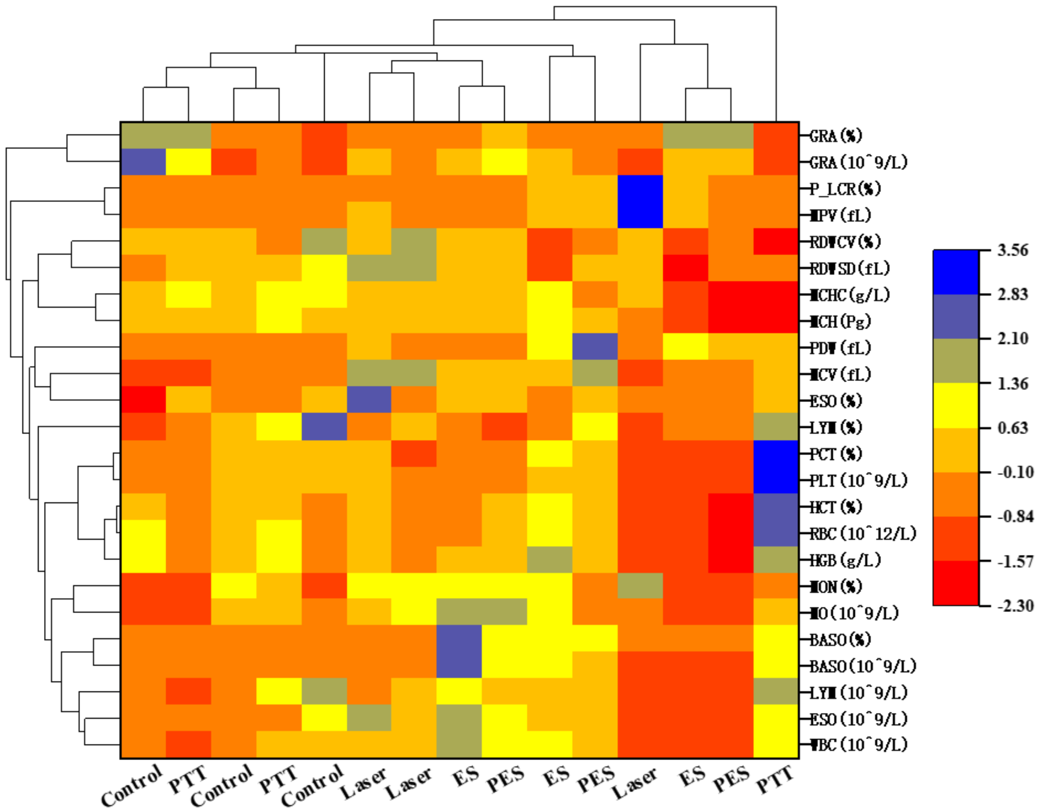

Supplementary Figure 29 │ Cluster analysis heat map of the hematological indexes of the tested mice in all groups.

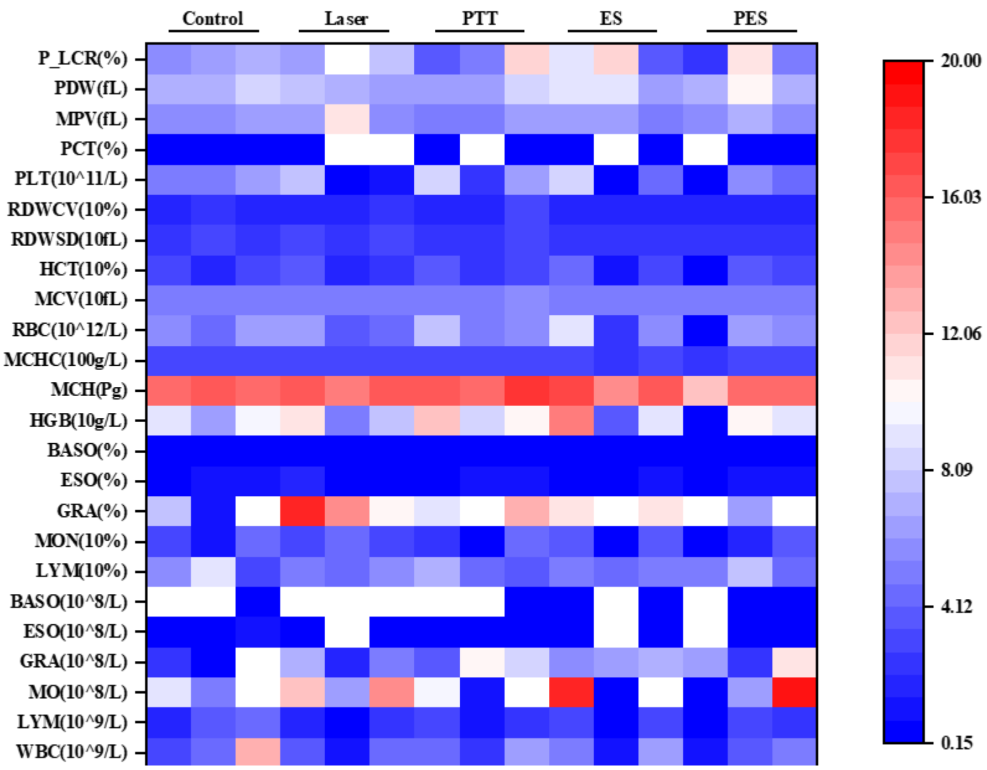

Supplementary Figure 30 │ Hematological indexes of mice euthanized after 15 days of tumor therapy in all groups.

Supplementary Table 1: Primer pairs used for PCR.

PCR primers	Sequence 5'-3'	Reference
Caspase-3-F	GTGGAACTGACGATGATATGGC	5
Caspase-3-R	CGCAAAGTGACTGGATGAACC	5
GSDME-F	TGCAACTTCTAAGTCTGGTGACC	6
GSDME-R	CTCCACAACCACTGGACTGAG	6
IL-1β-F	TGAAATGCCACCTTTTGACAG	7
IL-1β-R	CCACAGCCACAATGAGTGATAC	7
Bax-F	CGGCGAATTGGAGATGAACTGG	5
Bax-R	CTAGCAAAGTAGAAGAGGGCAACC	5
Cyt-c-F	GGCTGCTGGATTCTCTTACACA	7
Cyt-c-R	CCAAATACTCCATCAGGGTATCCT	7
c-Jun-F	TTCCTCCAGTCCGAGAGCG	6
c-Jun-R	TGAGAAGGTCCGAGTTCTTGG	6

Supplementary Table 2: The weight of mice body and tumors in different groups (unit: g)

Control		Laser		PTS		ES		PES	
Body weight	Tumor weight	Body weight	Tumor weight	Body weight	Tumor weight	Body weight	Tumor weight	Body weight	Tumor weight
21.43	1.72	21.11	1.9	21.11	1.45	21.76	1.6	19.43	0.1
23.24	2.04	20.06	1.44	19.63	1.16	19.04	1.16	19.21	0.4
20.05	1.73	23.56	2.1	19.95	1.06	21.11	1.43	19.11	0.86

Supplementary Table 3: The absolute tumor volumes in different groups (unit: mm^3)

Tumor volumes				
Control	Laser	PTS	ES	PES
1815.94	1789.79	1579.44	1497.06	229.65
1664.05	1690.59	1308.14	1535.35	181.59
1860.08	1821.66	1351.79	1461.50	176.45

* A full set of data of the tumor volumes for all the examined mice and every measurement:

Control:

Mice \ Day	0	2	4	6	8	10	12	14	15
**1**	99.95	123.34	217.10	391.48	459.65	932.71	1217.40	1612.12	1815.94
**2**	100.48	127.94	198.95	329.87	438.31	789.75	937.35	1446.13	1664.05
**3**	99.93	124.15	182.36	393.51	583.05	999.68	1321.87	1685.87	1860.08
**4**	97.19	114.32	168.56	356.82	478.09	768.97	1051.31	1643.81	1772.12

Laser:

Mice \ Day	0	2	4	6	8	10	12	14	15
**1**	100.26	154.14	230.27	365.49	499.48	805.86	1092.91	1598.51	1798.79
**2**	100.17	108.48	161.30	285.99	505.01	609.28	856.30	1513.90	1690.59
**3**	99.47	106.83	142.72	206.17	398.13	651.45	862.24	1459.14	1693.98
**4**	98.88	130.87	219.88	375.33	602.61	831.73	1036.78	1668.88	1821.66

PTS:

Mice \ Day	0	2	4	6	8	10	12	14	15
**1**	100.60	142.19	204.94	291.87	592.83	858.33	1048.72	1492.38	1579.44
**2**	100.81	143.46	275.01	484.93	646.94	803.54	1063.59	1202.90	1308.14
**3**	100.31	129.27	196.75	306.70	597.06	803.46	1019.76	1209.81	1351.79
**4**	100.13	117.81	155.25	279.62	390.67	706.10	1087.55	1285.64	1374.17

ES:

Mice \ Day	0	2	4	6	8	10	12	14	15
**1**	99.27	127.62	140.50	281.19	417.83	707.37	1188.17	1402.72	1529.51
**2**	99.91	103.97	132.10	199.88	304.45	640.15	874.25	1201.39	1461.50
**3**	100.18	144.46	236.55	348.00	685.73	902.74	1306.31	1469.99	1535.35
**4**	99.22	138.98	178.67	261.65	531.51	863.08	1175.22	1407.03	1497.06

PES:

Mice \ Day	0	2	4	6	8	10	12	14	15
**1**	100.13	177.94	197.41	197.91	206.47	210.63	229.39	223.76	229.65
**2**	99.05	107.61	148.29	190.12	184.32	180.66	182.59	182.46	181.59
**3**	100.39	108.54	147.53	166.05	163.77	177.25	175.61	175.29	176.45
**4**	97.95	100.43	101.39	108.91	120.88	124.63	123.12	126.28	128.18

Supplementary References:

1. Alhabeb, M. et al. Guidelines for synthesis and processing of two-dimensional titanium carbide ($Ti_3C_2T_x$ MXene). Chem. Mater. **29**, 7633-7644 (2017).

2. Ding, X. et al. Surface plasmon resonance enhanced light absorption and photothermal therapy in the second near-infrared window. J. Am. Chem. Soc. **136**, 15684-15693 (2014).

3. Chen, G. et al. Glutamine antagonist synergizes with electrodynamic therapy to induce tumor regression and systemic antitumor immunity. ACS Nano **16,** 951-962 (2022).

4. Tong X, et al. Targeting cell death pathways for cancer therapy: recent developments in necroptosis, pyroptosis, ferroptosis, and cuproptosis research. J. Hematol. Oncol. **15**, 174 (2022).

5. Li, X. et al. Effect of chitooligosaccharides on cyclin D1, bcl-xl and bcl-2 mRNA expression in A549 cells using quantitative PCR. Chin. Sci. Bull. **56**, 1629-1632 (2011).

6. Wang, Y. et al. Chemotherapy drugs induce pyroptosis through caspase-3 cleavage of a gasdermin. Nature **547**, 99-103 (2017).

7. Zhang, X. et al. Differential IL18 signaling via IL18 receptor and Na-Cl co-transporter discriminating thermogenesis and glucose metabolism regulation. Nat. Commun. **13**, 7582 (2022).
