# Supplementary material for: A wearable electrostimulation-augmented ionic-gel photothermal patch doped with MXene for skin tumor treatment
Source: Nat Commun. 2024 Jan 26;15:762. doi: 10.1038/s41467-024-45070-z (PMC10817919; doi:10.1038/s41467-024-45070-z)
Supplement: Supplementary file 2 — Reporting Summary [file 41467_2024_45070_MOESM2_ESM.pdf]

nature portfolio

Corresponding author(s): Yongdong Jin

Last updated by author(s): Dec 25, 2023

Reporting Summary

Nature Portfolio wishes to improve the reproducibility of the work that we publish. This form provides structure for consistency and transparency in reporting. For further information on Nature Portfolio policies, see our Editorial Policies and the Editorial Policy Checklist.

Statistics

For all statistical analyses, confirm that the following items are present in the figure legend, table legend, main text, or Methods section.

n/a	Confirmed	
☐	☒	The exact sample size (n) for each experimental group/condition, given as a discrete number and unit of measurement
☒	☐	A statement on whether measurements were taken from distinct samples or whether the same sample was measured repeatedly
☐	☒	The statistical test(s) used AND whether they are one- or two-sided<br>*Only common tests should be described solely by name; describe more complex techniques in the Methods section.*
☒	☐	A description of all covariates tested
☒	☐	A description of any assumptions or corrections, such as tests of normality and adjustment for multiple comparisons
☐	☒	A full description of the statistical parameters including central tendency (e.g. means) or other basic estimates (e.g. regression coefficient) AND variation (e.g. standard deviation) or associated estimates of uncertainty (e.g. confidence intervals)
☐	☒	For null hypothesis testing, the test statistic (e.g. F, t, r) with confidence intervals, effect sizes, degrees of freedom and P value noted<br>*Give P values as exact values whenever suitable.*
☒	☐	For Bayesian analysis, information on the choice of priors and Markov chain Monte Carlo settings
☒	☐	For hierarchical and complex designs, identification of the appropriate level for tests and full reporting of outcomes
☐	☒	Estimates of effect sizes (e.g. Cohen's d, Pearson's r), indicating how they were calculated

Our web collection on statistics for biologists contains articles on many of the points above.

Software and code

Policy information about availability of computer code

Data collection	Data were collected using the software that came with the instrument.
Data analysis	We used OriginPro 2021b (64-bit) SR1 9.8.5.204 (Education Edition) and imageJ Version 1.51.

For manuscripts utilizing custom algorithms or software that are central to the research but not yet described in published literature, software must be made available to editors and reviewers. We strongly encourage code deposition in a community repository (e.g. GitHub). See the Nature Portfolio guidelines for submitting code & software for further information.

Data

Policy information about availability of data

All manuscripts must include a data availability statement. This statement should provide the following information, where applicable:

- Accession codes, unique identifiers, or web links for publicly available datasets
- A description of any restrictions on data availability
- For clinical datasets or third party data, please ensure that the statement adheres to our policy

The main data supporting the results in this study are available within the paper and its Supplementary Information. All raw and analyzed datasets generated during the study are available for research purposes from the corresponding authors on request.

Research involving human participants, their data, or biological material

Policy information about studies with human participants or human data. See also policy information about sex, gender (identity/presentation), and sexual orientation and race, ethnicity and racism.

Reporting on sex and gender	N/A
Reporting on race, ethnicity, or other socially relevant groupings	N/A
Population characteristics	N/A
Recruitment	N/A
Ethics oversight	N/A

Note that full information on the approval of the study protocol must also be provided in the manuscript.

Field-specific reporting

Please select the one below that is the best fit for your research. If you are not sure, read the appropriate sections before making your selection.

☒ Life sciences ☐ Behavioural & social sciences ☐ Ecological, evolutionary & environmental sciences

For a reference copy of the document with all sections, see nature.com/documents/nr-reporting-summary-flat.pdf

Life sciences study design

All studies must disclose on these points even when the disclosure is negative.

Sample size	The numher of animals in each groun was determined according to previous studies cited in our manuscrint The size of each sample is in close agreement with those studies already published. Sample sizes for each experiment were described in figure legends. For all experiments in this study, n ≥ 3.
Data exclusions	No data were excluded from the analyses.
Replication	Three parallel sets of experiments were performed for each experiment, and the results obtained were reproducible.
Randomization	Randomly classified according to the size of the tumor generated before treatment (mean tumor volume was the same in each group).
Blinding	Our experiment was a single-blind experiment, and the experimental subjects were mice. The experimentalists knew the condition of the subjects and the experimental conditions.

Reporting for specific materials, systems and methods

We require information from authors about some types of materials, experimental systems and methods used in many studies. Here, indicate whether each material, system or method listed is relevant to your study. If you are not sure if a list item applies to your research, read the appropriate section before selecting a response.

Materials & experimental systems

n/a	Involved in the study	
☐	☒	Antibodies
☐	☒	Eukaryotic cell lines
☒	☐	Palaeontology and archaeology
☐	☒	Animals and other organisms
☒	☐	Clinical data
☒	☐	Dual use research of concern
☐	☒	Plants

Methods

n/a	Involved in the study	
☒	☐	ChIP-seq
☒	☐	Flow cytometry
☒	☐	MRI-based neuroimaging

Antibodies

Antibodies used	Anti-γH2A.X antibody was purchased from Abcam, catalog number is ab11174, clone name is rabbit polyclonal antibody to gamma H2A.X (phospho S139). Anti -Ki67 Mouse mAb was purchased from Servicebio, catalog number is GB121141, clone name is rabbit polyclonal antibody. Anti -Cleaved- Caspase-3 Rabbit pAb was purchased from Servicebio, catalog number is GB11532, clone name is rabbit polyclonal antibody. Anti-DFNA5/GSDME[EPR19859] - N-terminal was purchased from Abcam, atalog number is ab215191,

	clone name is rabbit polyclonal antibody.
Validation	All the antibodies used in this study are commercially available. The information of these antibodies are available in the manufacture's website.<br>Anti-γH2A.X: https://www.abcam.cn/products/primary-antibodies/gamma-h2ax-phospho-s139-antibody-ab11174.html<br>Anti -Ki67: https://www.servicebio.cn/search-result?search=GB121141<br>Anti-DFNA5/GSDME[EPR19859] - N-terminal: https://www.abcam.com/products/primary-antibodies/dfna5gsdme-antibody-epr19859-n-terminal-ab215191.html

Eukaryotic cell lines

Policy information about cell lines and Sex and Gender in Research

Cell line source(s)	The melanoma cells (B16F10) were obtained from the American Type Culture Collection (ATCC, USA). Derived from C57BL/6J mouse spontaneous tumor cells. The sex of the B16F10 cell is male.
Authentication	DNA was extracted with Axygen's genome extraction kit and amplified with 10-STR amplification protocol. The STR locus and sex gene Amelogenin were detected on ABI 3730XL genetic analyzer.
Mycoplasma contamination	All cell lines tested negative for mycoplasma contamination.
Commonly misidentified lines<br>(See ICLAC register)	The cell line was identified and there was no misuse of the cell line.

Animals and other research organisms

Policy information about studies involving animals; ARRIVE guidelines recommended for reporting animal research, and Sex and Gender in Research

Laboratory animals	C57BL/6J mice (4 weeks old, female) were purchased from Beijing HFK Biotechnology Ltd (Beijing, China). The living environment of animals was maintained at a temperature of 25℃ and at 40–70% humidity with a 12 h light/dark cycle, with free access to standard food and water.
Wild animals	The study did not involve wild animals.
Reporting on sex	All the mice were female, which could control the experimental variables and prevent unnecessary deaths from male fights later in the culture.
Field-collected samples	Mouse samples were collected under sterile conditions of 25 degrees Celsius. No field collected samples were used in the study.
Ethics oversight	All mice were raised and cared according to the guidelines on Laboratory Animals of Jilin University. Meanwhile, all animal procedure was approved by the Laboratory Animal Management Committee of Jilin University. Pork was obtained from a local market (no live animals were involved).

Note that full information on the approval of the study protocol must also be provided in the manuscript.

Dual use research of concern

Policy information about [dual use research of concern]

Hazards

Could the accidental, deliberate or reckless misuse of agents or technologies generated in the work, or the application of information presented in the manuscript, pose a threat to:

No	Yes	
x		Public health
x		National security
x		Crops and/or livestock
x		Ecosystems
x		Any other significant area

Experiments of concern

Does the work involve any of these experiments of concern:

No	Yes	
x		Demonstrate how to render a vaccine ineffective
x		Confer resistance to therapeutically useful antibiotics or antiviral agents
x		Enhance the virulence of a pathogen or render a nonpathogen virulent
x		Increase transmissibility of a pathogen
x		Alter the host range of a pathogen
x		Enable evasion of diagnostic/detection modalities
x		Enable the weaponization of a biological agent or toxin
x		Any other potentially harmful combination of experiments and agents
